# Metformin-induced preferential killing of breast cancer initiating CD44^+^CD24^−/low^ cells is sufficient to overcome primary resistance to trastuzumab in HER2+ human breast cancer xenografts

**DOI:** 10.18632/oncotarget.488

**Published:** 2012-05-04

**Authors:** Sílvia Cufí, Bruna Corominas-Faja, Alejandro Vazquez-Martin, Cristina Oliveras-Ferraros, Joan Dorca, Joaquim Bosch-Barrera, Begoña Martin-Castillo, Javier A. Menendez

**Affiliations:** ^1^ Translational Research Laboratory, Catalan Institute of Oncology (ICO), Girona, Spain; ^2^ Girona Biomedical Research Institute (IDIBGi), Girona, Spain; ^3^ Medical Oncology, Catalan Institute of Oncology (ICO), Girona, Spain; ^4^ Clinical Research Unit, Catalan Institute of Oncology (ICO), Girona, Spain

**Keywords:** Metformin, trastuzumab, HER2, cancer stem cells, breast cancer

## Abstract

Trastuzumab-refractory breast cancer stem cells (CSCs) could explain the high rate of primary resistance to single-agent trastuzumab in *HER2* gene-amplified breast cancer patients. The identification of agents with strong selective toxicity for trastuzumab-resistant breast CSCs may have tremendous relevance for how HER2+ breast cancer patients should be treated. Using the human breast cancer cell line JIMT-1, which was established from the pleural metastasis of a patient who was clinically resistant to trastuzumab *ab initio*, we examined whether preferential killing of the putative CD44^+^CD24 ^−/low^ breast CSC population might be sufficient to overcome primary resistance to trastuzumab *in vivo*. Because recent studies have shown that the anti-diabetic biguanide metformin can exert antitumor effects by targeted killing of CSC-like cells, we explored whether metformin's ability to preferentially kill breast cancer initiating CD44^+^CD24 ^−/low^ cells may have the potential to sensitize JIMT-1 xenograft mouse models to trastuzumab. Upon isolation for breast cancer initiating CD44^+^CD24 ^−/low^ cells by employing magnetic activated cell sorting, we observed the kinetics of metformin-induced killing drastically varied among CSC and non-CSC subpopulations. Metformin's cell killing effect increased dramatically by more than 10-fold in CD44^+^CD24 ^−/low^ breast CSC cells compared to non-CD44^+^CD24 ^−/low^ immunophenotypes. While seven-weeks treatment length with trastuzumab likewise failed to reduce tumor growth of JIMT-1 xenografts, systemic treatment with metformin as single agent resulted in a significant two-fold reduction in tumor volume. When trastuzumab was combined with concurrent metformin, tumor volume decreased sharply by more than four-fold. Given that metformin-induced preferential killing of breast cancer initiating CD44^+^CD24 ^−/low^ subpopulations is sufficient to overcome *in vivo* primary resistance to trastuzumab, the incorporation of metformin into trastuzumab-based regimens may provide a valuable strategy for treatment of HER2+ breast cancer patients.

## INTRODUCTION

*De novo* (*i. e.* primary) resistance to the monoclonal antibody trastuzumab (Herceptin) remains a prevalent challenge in the treatment of breast cancer patients whose tumors overexpress the human epidermal growth factor 2 (HER2) [1]. Evidence is mounting that the CD44^+^CD24^−/low^ cell subpopulation, which is enriched with potential breast cancer stem cells (CSCs), could explain clinical resistance to HER2-targeted therapies [1-3]. Therefore, future clinical trials should involve the integration of novel anti-breast cancer stem cells approaches to prevent and overcome the inherent unresponsiveness to trastuzumab across clinically important subgroups of HER2-positive breast cancer patients. Although *in vitro* studies have recently suggested that the anti-diabetic drug metformin can efficiently eliminate treatment-resistant stem/progenitor cell populations in heterogeneous breast cancer populations [4-6], it remained to be evaluated whether systemic metformin might overcome primary resistance to trastuzumab in *HER2*-gene amplified human breast cancer xenografts. Here we explored for the first time the *in vivo* sensitizing efficacy of metformin on trastuzumab therapy by using xenografts of the human breast cancer cell line JIMT-1, which was established from the pleural metastasis of a patient who was clinically resistant to trastuzumab *ab initio* [7]. The JIMT-1 model is unique because of displaying at the same time several co-existing mechanisms of resistance to trastuzumab present at variable levels in other breast cancer cell lines, including moderate expression levels of HER2 (despite *HER2* gene amplification), low expression of PTEN (phosphatase and tensin homolog), an activating mutation of the *PIK3CA* gene, high expression of NRG1 (neuregulin-1), and enhanced expression of mesenchymal markers, including a naturally enriched subpopulation of breast cancer initiating CD44^+^CD24^−/low^ CSC-like cells [2, 8-10].

## RESULTS AND DISCUSSION

### Metformin preferentially kills breast cancer initiating CD44^+^CD24^−^/low cells

**Figure 1:**
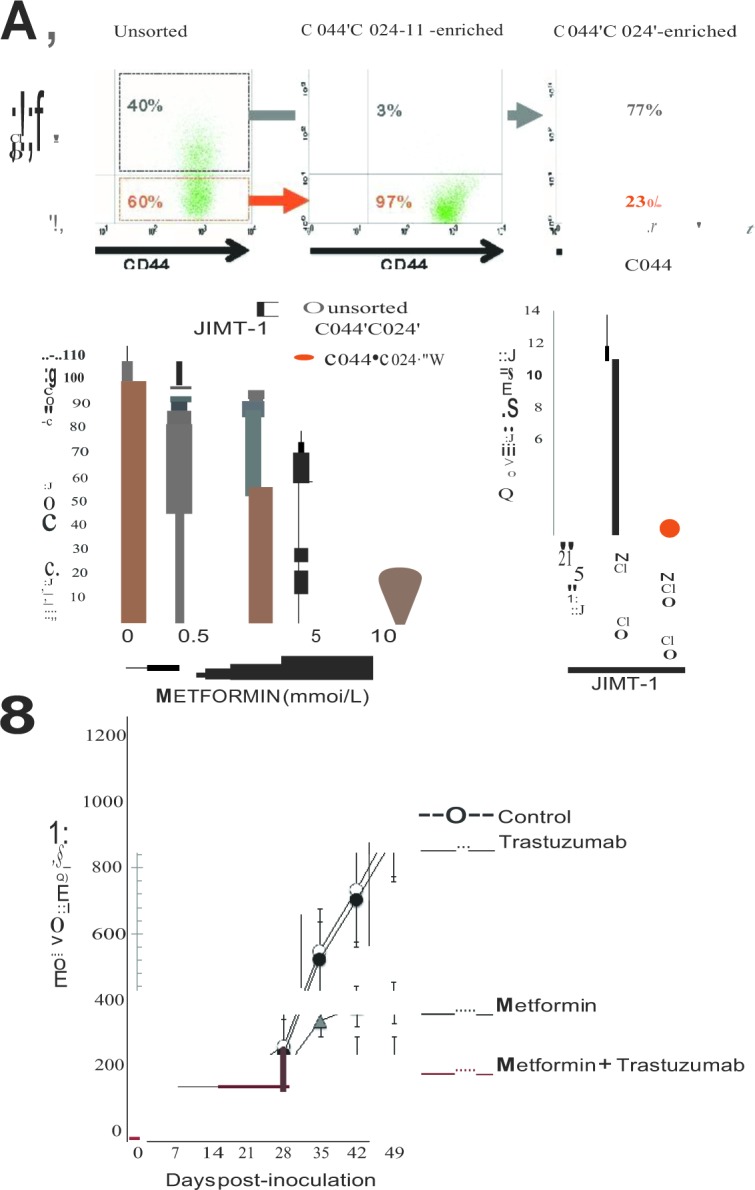
**A.** *Top*. CD44 and CD24 expression patterns in CD44^+^CD24^−/low^ and CD44^+^CD24^+^ subpopulations of JIMT-1 sorted by sequential sorting (depletion followed by positive selection) using MACS MicroBeads (MACS^®^ Technology). Enrichment of target cells by magnetic MicroBeads was carried out according to the manufacturer’s (Milteny Biotec, Bergisch Gladbach, Germany) protocol. CD44^+^CD24^−/low^ were isolated from the parental JIMT-1 cell line by firstly depleting CD24^+^ cells using the CD24 MicroBead Kit and then positively selected for CD44 using the CD44 MicroBeads. Cells were fluorescently stained with combinations of fluorochrome-conjugated monoclonal antibodies obtained from BD Biosciences (San Diego, CA, USA) against human CD44 (FITC; cat.#555478) and CD24 (PE; cat.#555428) or their respective isotype controls. Representative expression (n=5) in pre-sorted and post-sorted JIMT-1 cells is shown. *Bottom*. Parental JIMT-1 cells and cells sorted for BCSC^+^CD44^+^CD24^−/low^ and non-BCSC^−^CD44^+^CD24^+^ markers were treated simultaneously with increasing concentrations of metformin for 5 days. Cell viability was determined using a standard colorimetric MTT (3-4, 5-dimethylthiazol-2-yl-2, 5-diphenyl-tetrazolium bromide) reduction assay. For each treatment, cell viability was evaluated as a percentage using the following equation: (OD_570_ of treated sample/OD_570_ of untreated sample) × 100. Cell sensitivity to metformin was expressed in terms of the concentration of drug required to decrease by 50% cell viability (IC_50_ value). Since the percentage of control absorbance was considered to be the surviving fraction of cells, the IC_50_ values were defined as the concentration of agents that produced 50% reduction in control absorbance. B. Tumor xenografts were established by subcutaneous injection of 5 × 10^6^ JIMT-1 cells suspended in 100 μl of culture medium into the flank of female athymic nude mice (four to five weeks old, 23 to 25 g; Harlan Laboratories —France-). Animals were randomized into four groups with five mice in each group: control (vehicles), trastuzumab, metformin, and trastuzumab + Metformin. Trastuzumab (5 mg/kg) was given intraperitoneally (i.p.) once per week. Both the “metformin group” and the “trastuzumab + metformin group” received daily a single i.p. injection of metformin (250 mg/kg). Mice were weighed once per week after dosing, tumors were measured daily with electronic callipers, and tumor volumes were calculated using the following formula: volume (mm^3^) = length *x* width^2^ *x* 0.5. Figure shows mean tumor volumes (±SD) of JIMT-1 xenograft-bearing nude mice following injection with trastuzumab, metformin, and trastuzumab + metformin until seven weeks.

We first examined whether breast cancer initiating CD44^+^CD24^−/low^ and non-CD44^+^CD24^−/low^ cell subpopulations from the trastuzumab-refractory JIMT-1 cell line exhibited differential sensitivities to the growth inhibitory effects of metformin. We employed MTT-based cell viability assays to compare the degree of sensitivity to metformin of parental JIMT-1 cells and that of JIMT-1 cell subpopulations isolated and purified for breast cancer initiating CD44^+^CD24^−/low^ and non-CD44^+^CD24^−/low^ immunophenotypes by employing magnetic activated cell sorting (MACS; Fig. 1A, *top panels*). Cells were treated side-by-side on the following sorting day with graded concentrations of metformin for five days. While unsorted JIMT-1, CD44^+^CD24^−/low^, and CD44^+^CD24^+^ populations all showed an inhibitory response to metformin that augmented with the increase in metformin concentration (Fig. 1A, *bottom left*), each separate cell population differed in their extend of cytotoxic response to metformin. The breast cancer initiating CD44^+^CD24^−/low^ subpopulation was significantly more sensitive than was the parental or CD44^+^CD24^+^ populations. CD44^+^CD24^−/low^ cells had an estimated IC_50_ value of 1±0.2 mmol/L metformin whereas that in the corresponding non-CD44^+^CD24^−/low^ population was 11±2 mmol/L metformin (Fig. 1A, *bottom right*). The IC_50_ of metformin in the unsorted JIMT-1 parental population was 8±2 mmol/L (Fig. 1A, *bottom right*). These findings, altogether, confirmed that the breast cancer initiating subpopulation within a heterogeneous population of trastuzumab-refractory, HER2-positive breast cancer cells notably exhibit greater sensitivity to the growth-inhibitory effects of metformin. Indeed, the growth of the breast cancer initiating CD44^+^CD24^−/low^ population was more significantly affected by metformin at all tested concentrations. Because it has been shown that gradual loss of stem cell markers takes place in cells growing as a monolayer, these data may further underestimate hypersensitivity of the breast cancer initiating CD44^+^CD24^−/low^ cells to metformin. Indeed, JIMT-1 cells sorted for the CD44^+^CD24^−/low^ immunophenotype repopulated all the parental cell fractions after few days in monolayer culture (data not shown).

### Systemic metformin inhibits tumor growth in trastuzumab-refractory breast cancer xenografts

The effects of metformin and trastuzumab alone and in combination on tumor growth were next studied *in vivo* using a JIMT-1 xenograft animal model (Fig. 1B). Compared to the control group after seven weeks of treatment (940±170 mm^3^), the trastuzumab-treated group likewise failed to exhibit significant reductions in mean tumor size (891±135 mm^3^). Compared to the mean xenograft tumor size in both the control and the trastuzumab group, the mean tumor size in the metformin group was significantly smaller (390±64 mm^3^), which confirmed that the inhibitory effect of metformin at the tested concentration (250 mg/kg/day) was notably stronger than that of trastuzumab (5 mg/kg/week) on JIMT-1 tumor growth. When the two drugs were combined, the xenograft tumor size (213±75 mm^3^) was smaller than those of the groups treated with trastuzumab or metformin alone, indicating that the combination of the drugs was much more effective at reducing tumor volume. Consequently, the days required for four-fold increase in tumor volume was 27±5 after treatment with trastuzumab whereas more than 50 days were required after the combined treatment with trastuzumab and metformin (Fig. 1B). No significant difference in body weight was observed in xenograft tumor-bearing mice between the treatment groups (data not shown).

Our current findings reveal that: a.) Metformin as single agent is active against JIMT-1-derived tumor xenografts with primary resistance to trastuzumab; b.) metformin’s ability to distinctively kill breast cancer initiating CD44^+^CD24^−/low^ mesenchymal subpopulations is sufficient to overcome *in vivo* primary resistance to trastuzumab in HER2-gene amplified breast tumors. A combination of trastuzumab and metformin may provide a valuable strategy for treatment of HER2-overexpressing breast cancer, and a phase II trial recently has opened in Spain to evaluate the efficacy of trastuzumab plus metformin as neo-adjuvant therapy for patients with HER2-positive breast cancer [11].
